# Postoperative delayed massive bleeding in gastric cancer: a case report

**DOI:** 10.1186/s13256-024-04531-1

**Published:** 2024-04-25

**Authors:** Zhongting Lu, Chenhui Qin, Mingxuan Zhang, Tao Li

**Affiliations:** 1 Graduate School, Ningxia Medical University, Ningxia, China; 2 The General Hospital of Taiyuan Central Hospital, Shanxi, China; 3 Department of Surgical Oncology, Tumor Hospital, The General Hospital of Ningxia Medical University, Ningxia, China

**Keywords:** Gastric malignancy, Postoperative complications, Hemorrhage, Surgical interventions, Case report

## Abstract

**Background:**

Postoperative delayed bleeding of gastric cancer is a complication of radical gastrectomy with low incidence rate and high mortality.

**Case presentation:**

This case report presents the case of a 63-year-old female patient of Mongolian ethnicity who was diagnosed with gastric malignancy during a routine medical examination and underwent Billroth's I gastric resection in our department. However, on the 24th day after the surgery, she was readmitted due to sudden onset of hematemesis. Gastroscopy, abdominal CT, and digital subtraction angiography revealed postoperative anastomotic fistula, rupture of the duodenal artery, and bleeding from the abdominal aorta. The patient underwent three surgical interventions and two arterial embolizations. The patient’s condition stabilized, and she was discharged successfully.

**Conclusion:**

Currently, there are no specific guidelines for the diagnosis and treatment of pseudoaneurysms in the abdominal cavity resulting from gastric cancer surgery. Early digital subtraction angiography examination should be performed to assist in formulating treatment plans. Early diagnosis and treatment contribute to an improved overall success rate of rescue interventions.

## Introduction

Postoperative bleeding in gastric cancer carries a high risk and can lead to poor prognosis, prolonged hospitalization, increased financial burden for patients, and other complications [1, 2]. Delayed bleeding, typically occurring more than 24 h after surgery, is considered a late complication [3, 4]. In rare cases, bleeding may occur even more than one month postoperatively, often involving the intra-abdominal or gastrointestinal regions. Due to its unpredictable timing, inconspicuous location, slow bleeding rate, and relatively small volume, delayed bleeding poses challenges, particularly when it occurs after discharge, making timely intervention difficult. Herein, we present a case report of a patient who experienced sudden hematemesis 24 days after gastric cancer surgery.

## Case presentation

**Fig. 1:**
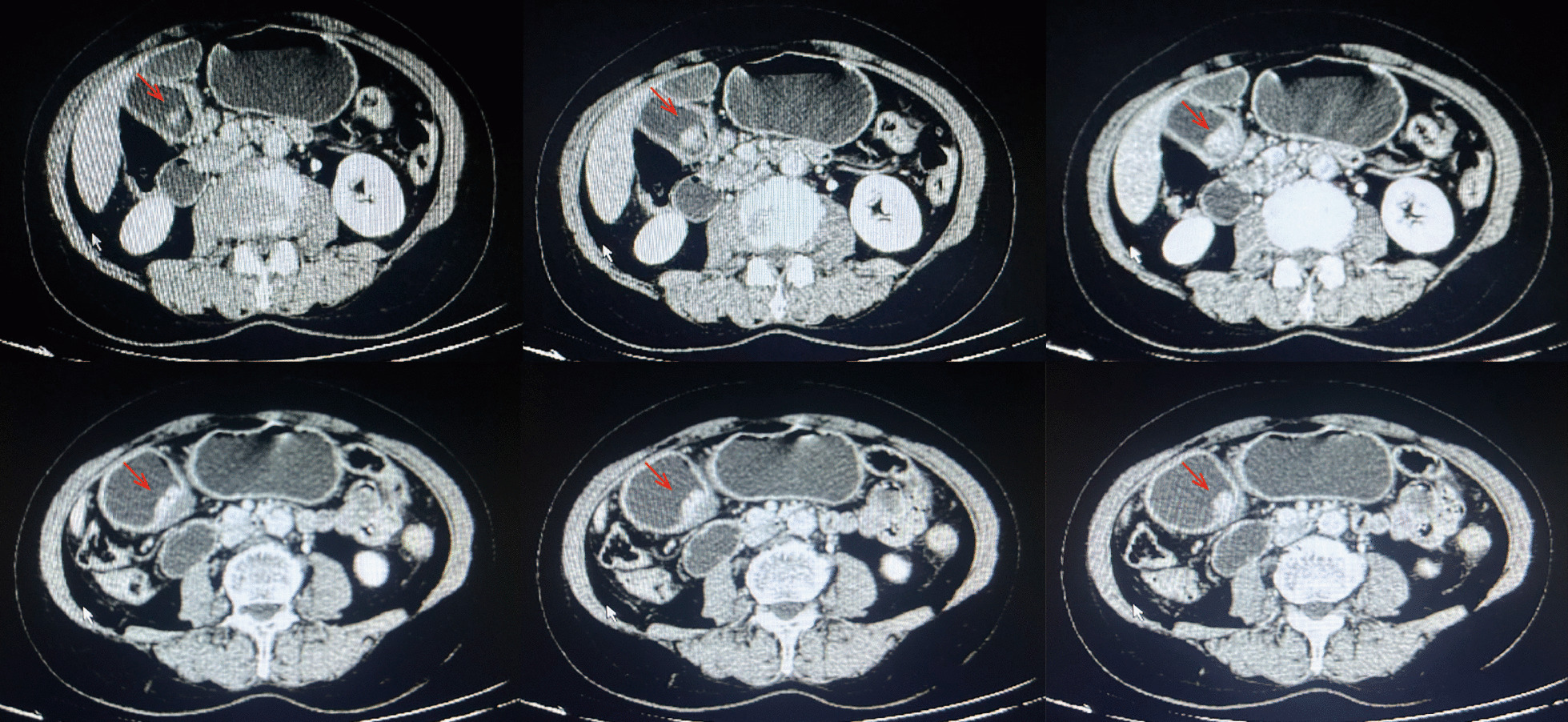
Gastric Computed tomography revealed mucosal thickening and ulceration in the gastric antrum, with para-antrum lymph node metastasis(arrows)

A 63-year-old female patient of Mongolian ethnicity, without any family history of hereditary diseases, was admitted to our department for treatment of gastric antral carcinoma detected by physical examination.She had a history of hypertension for 6 years, which was poorly controlled despite regular oral medication with candesartan. At presentation, physical examination showed stable vital signs and no obvious abnormalities in abdominal and other physical examinations. Gastric CT showed thickening of the antrum mucosa, visible ulcer formation, clear boundary between the lesion and the surrounding normal gastric wall, and enlarged lymph nodes near the antrum (Fig. 1). One week later, she underwent a “Laparoscopic D2 Modified Radical Gastrectomy for Gastric Cancer” without requiring blood transfusion. The postoperative pathological diagnosis revealed signet ring cell carcinoma of the gastric antrum (Lauren classification: diffuse type) with a maximum tumor diameter of 2.4 cm, infiltrating the deep muscular layer. There was no evidence of vascular tumor embolus or neural invasion. Metastasis was observed in the right cardia lymph nodes and upper pyloric lymph nodes. Immunohistochemistry results showed CDX-2 (+), HER2 (0), E-Cad (+), CD31 (no intravascular tumor embolus), D2-40 (no lymphatic vessel tumor embolus), S-100 (no neural invasion), Ki-67 (60%), CK-Pan (+), and AB-PAS (+). The patient made a successful recovery and was discharged on the 12th postoperative day.

**Fig. 2:**
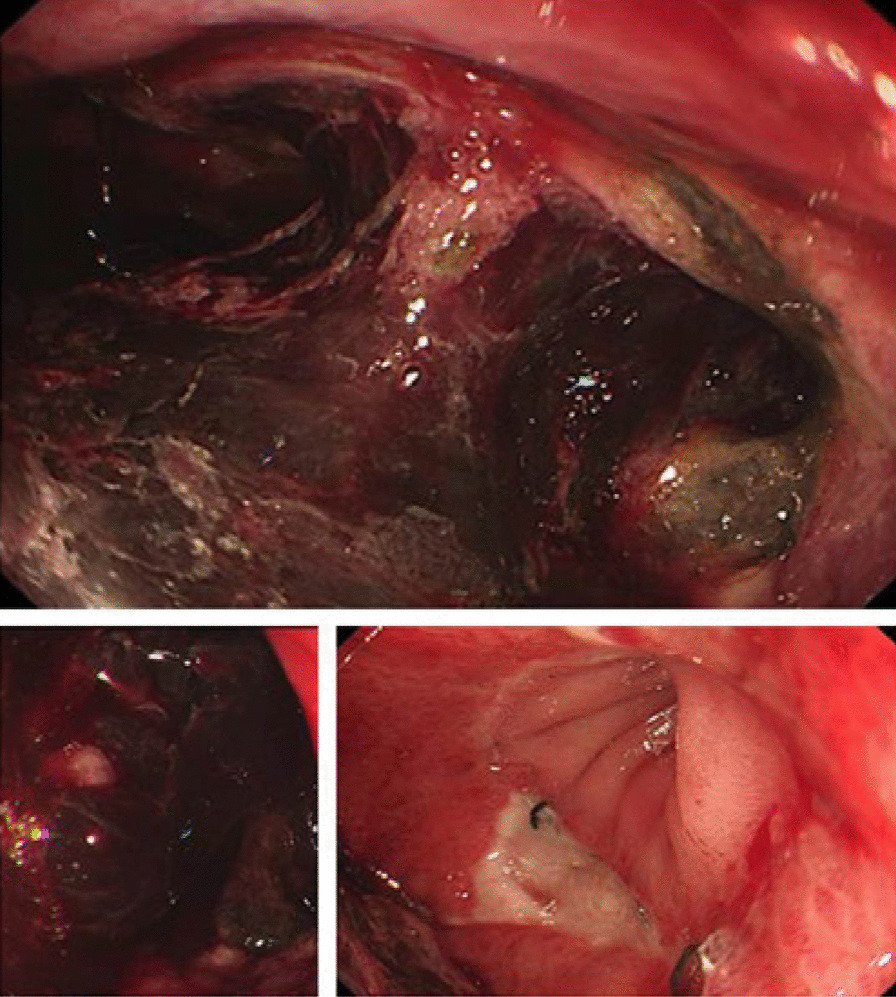
Esophagogastroduodenoscopy revealed ulcers on the surface of the remnant stomach, with a large number of blood clots in the center, and irregular ulcers at the anastomosis

On postoperative day 24, the patient was readmitted with sudden abdominal pain accompanied by hematemesis (approximately 300 ml) and fever (temperature reaching 39 °C). Physical examination revealed abdominal wall tension, a 5 cm scar in the midline of the abdomen, absence of gastric and intestinal peristalsis waves, tenderness localized to the upper abdomen, weakly positive rebound tenderness, no palpable masses, negative shifting dullness, and increased bowel sounds. Esophagogastroduodenoscopy showed an ulcerated surface in the residual gastric body with a large amount of blood clots attached centrally, no hematoma around the anastomosis site, irregular ulcers, no anastomotic stenosis, and unobstructed efferent loop (Fig. 2). Emergency “exploratory laparotomy and abdominal drainage” was performed. Intraoperatively, dense adhesions were observed in the peritoneum, and a small amount of fluid accumulation was seen in the abdominal cavity after blunt dissection, without gastric contents or blood clots. The residual gastric anterior wall was tightly adhered to the liver and transverse colon, with no evidence of residual gastric fistula or bleeding sites as indicated by the gastroscopy. No active bleeding was found during exploration, and 400 ml of blood was transfused intraoperatively. Postoperatively, a consultation with the nutrition department was requested to provide guidance on enteral and parenteral nutrition for the patient. The patient's condition remained stable, and there was an increase in hemoglobin levels compared to before the surgery.

On the 10th day after the first emergency surgery, the patient experienced sudden hematochezia with an estimated blood loss of approximately 400 ml. Considering the possibility of chronic intraperitoneal bleeding, a second emergency surgery was performed, involving “complete resection of the residual stomach and Roux-en-Y esophagojejunostomy”. Intraoperatively, exploration revealed necrotic perforation in the posterior wall of the residual stomach, with the bleeding point located near the gastric artery. Approximately 100 ml of blood was lost during the procedure, and 700 ml of packed red blood cells and 350 ml of plasma were transfused. The postoperative pathological examination indicated partial gastric tissue with focal dilated and congested blood vessels, necrosis, and hemorrhage within the gastric mucosa. Focal mucosal glandular hyperplasia and some glands showed low-grade epithelial dysplasia. The patient was transferred to a ward for intensified monitoring, and serial blood tests were conducted to assess the situation. Nutritional support was provided through parenteral routes.

On the 10th day after the second emergency surgery, the patient had dark red bloody drainage of approximately 800 ml from the right abdominal drain, with visible blood clots. To investigate the cause, a third emergency surgery was performed, involving “placement of a duodenal residual end tube and repair of esophagojejunostomy leakage”. Intraoperatively, the posterior wall of the esophagojejunostomy was found to be necrotic and ruptured, and a 3 mm leak was observed in the residual end of the duodenum. Active bleeding was located on the posterior wall of the esophagojejunostomy. Approximately 1700 ml of blood was lost during the procedure, and 2000 ml of blood and 800 ml of plasma were transfused. After three emergency surgeries, the patient was transferred to the special ward for intensified care. The patient had poor physical recovery due to the impact of the surgeries, and prolonged parenteral nutrition resulted in inadequate absorption of essential nutrients in the intestines.

**Fig. 3:**
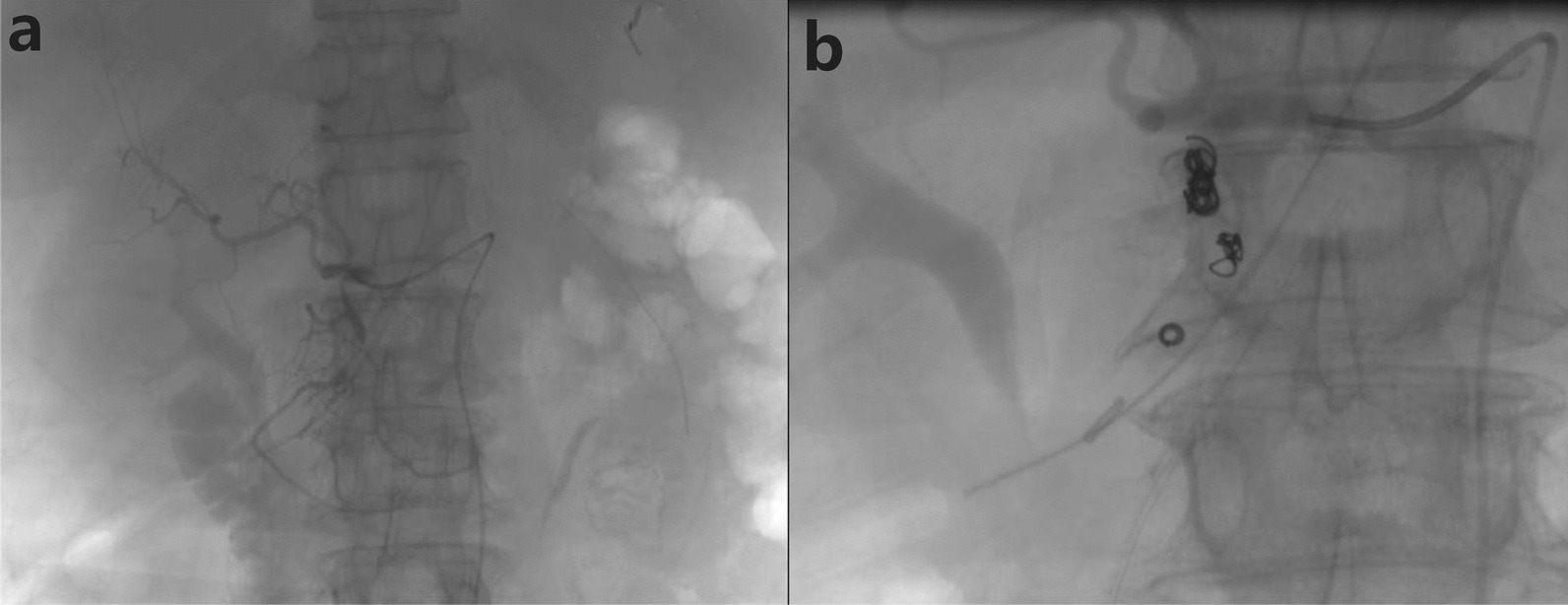
Angiography revealed hemorrhage from the superior mesenteric artery (**a**), and posterior duodenal artery embolization was performed (**b**)

**Fig. 4:**
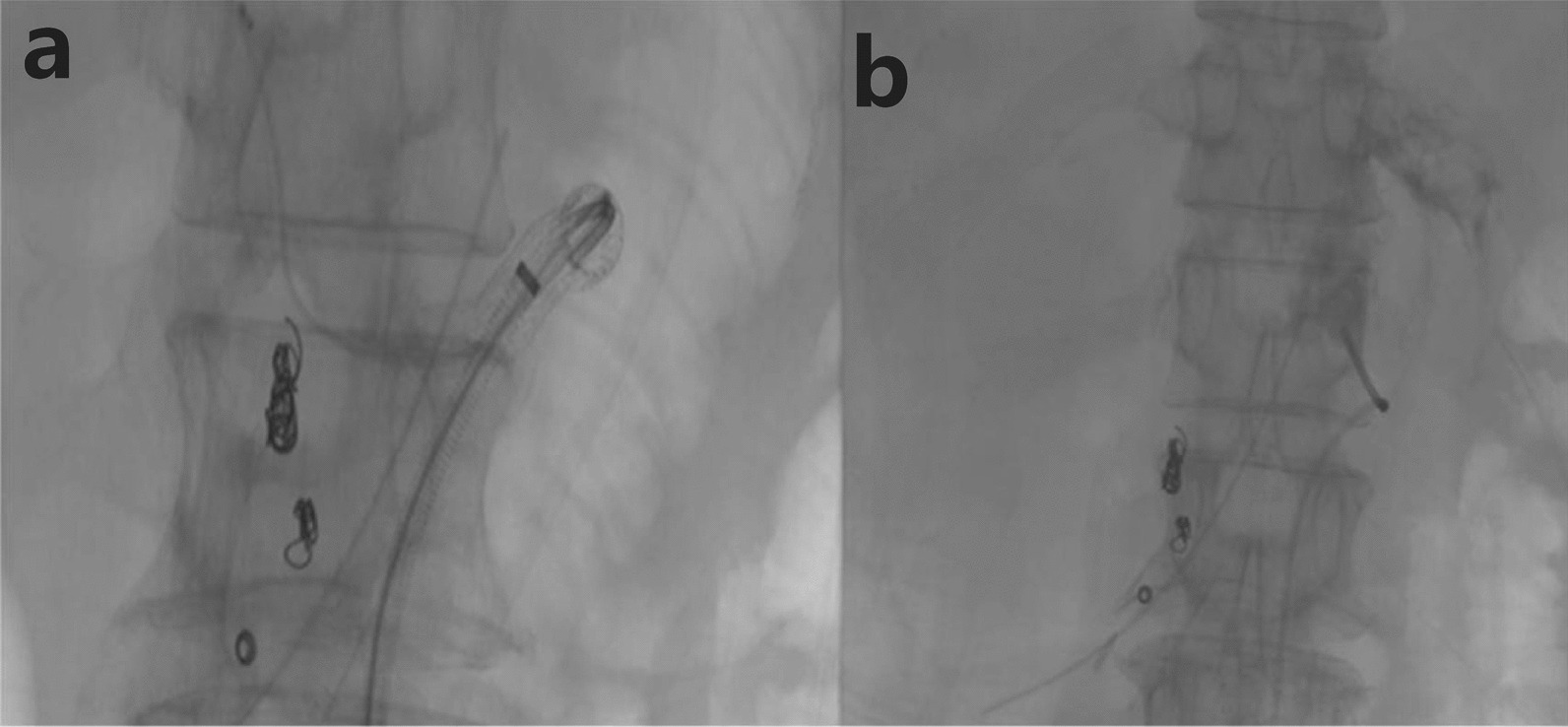
Angiography revealed contrast agent spillover in the proximal celiac trunk, but no contrast agent spillover at the original embolization of the duodenal artery (**a**). Celiac trunk artery stent implantation was performed (**b**)

On the 20th day after the third emergency surgery, there was another episode of intra-abdominal bleeding. A vascular surgery consultation was requested, and an emergency surgery was performed. Intraoperatively, angiography showed bleeding from the superior mesenteric artery, and “embolization of the posterior duodenal artery” was performed (Fig. 3). The procedure was successful. The next day, there was another episode of intra-abdominal bleeding. In two emergency surgeries performed by vascular surgery, extravasation of contrast agent was observed from the proximal segment of the abdominal aorta, and no extravasation was observed from the site of the original embolization of the superior mesenteric artery. “Placement of a stent in the abdominal aorta” was performed (Fig. 4), and after 10 min of observation, no blood was drained from the patient's drainage bag. The procedure was completed, and the patient returned to the ward. During the course of treatment, multiple consultations were requested from relevant departments to assist in diagnosis and treatment. After careful diagnosis and treatment, the patient’s condition stabilized, hemoglobin levels returned to normal, and the patient was able to ambulate. The patient’s nutrition was transitioned from parenteral to enteral feeding. One month later, the patient was discharged. Physical examination revealed well-fixed abdominal bandage, no tenderness or rebound tenderness in the abdomen, and no positive signs on further examination. The incision had healed, the right abdominal drain was unobstructed, draining serosanguinous fluid of 100 ml, and the duodenal T-tube was in place, draining brownish-yellow fluid of 40 ml.

After the patient's discharge, we conducted timely case discussions, and blood tests were performed before and after each surgery (Table 1). During the 1-month, 6-month, and 1-year follow-up visits after the surgery, there was no recurrent active bleeding at the surgical site, and the patient’s mental state was good. The diet primarily consisted of liquid food. Chemotherapy was initiated 1 year after the surgery.

**Table 1:** The blood routine examination of patient

Time	Preoperative			Postoperative		
	Hb	PLT	WBC	Hb	PLT	WBC
2018/12/1	102	263	11.51	104	125	3.68
2018/12/10	97	141	4.44	104	283	8.49
2018/12/20	95	456	10.71	104	125	3.68
2019/1/10	80	131	4.05	65	152	3.65
2019/1/11	50	85	7.82	129	315	5.13

Unit: Hb g/L, PLT × 10^9^/L, WBC × 10^9^/L

*Hb* Hemoglobin; *PLT* Platelet; *WBC* White Blood Cell

## Discussion

Delayed postoperative bleeding after gastric cancer surgery can be caused by multiple factors. How to reduce the occurrence of intra-abdominal and anastomotic bleeding after gastric cancer surgery while ensuring radical resection of the tumor has always been a hot topic of concern for scholars both domestically and internationally. Currently, the reported incidence of postoperative bleeding complications after radical gastric cancer surgery is 1% to 4% in the literature. Although the incidence is not high, it is associated with a relatively high mortality rate of approximately 10% to 20% [5–7]. One of the reasons for postoperative bleeding is rupture and hemorrhage of intra-abdominal vessels, including the left gastric artery, gastroduodenal artery, splenic artery, and abdominal aorta. The main reasons for these complications are excessive pursuit of vessel skeletonization during lymph node dissection, resulting in injury to the vascular system and surrounding nerve plexus, chronic electrocautery damage to the external membrane of the vessels during electrocautery procedures, local septicemia and infection due to perforation caused by chronic electrocautery damage to the external membrane or full-thickness injury, erosion of adjacent vessel walls by digestive juices in cases of leakage from the residual duodenum, inappropriate ligature of blood vessels leading to rupture and injury of the intima, and formation of pseudoaneurysms with subsequent rupture and hemorrhage [7–9]. The mortality rate associated with rupture of pseudoaneurysms after gastric cancer surgery has been reported to be over 50% [8].

Postoperative gastric acid secretion is generally reduced in patients with gastric cancer, and the use of postoperative acid-suppressing drugs further lowers the incidence of peptic ulcers [10]. In this case, the delayed postoperative bleeding in the patient was due to the formation and rupture of pseudoaneurysms. The patient had a history of poorly controlled hypertension, poor acid tolerance in the excluded jejunum, long-term chronic blood impact, and poor postoperative nutritional absorption. Sudden changes in blood pressure led to vascular rupture and anastomotic fistula, resulting in the influx of a large amount of digestive juice into the abdominal cavity, causing local septicemia. Under the influence of inflammation and trauma, pseudoaneurysms of the left gastric artery were formed. After partial gastrectomy, the patient developed esophagojejunostomy fistula and residual duodenal fistula, leading to the formation of pseudoaneurysms in the abdominal aorta and gastroduodenal artery.

Patient was readmitted with symptoms of abdominal pain and hematemesis of 300 ml, suggesting the presence of a bleeding point in the jejunum and rupture of the anastomosis. Post-gastric cancer surgery, intra-abdominal adhesions are prone to occur, and the influx of blood and digestive juices into the abdominal cavity worsens the adhesions. Conservative treatment is not feasible due to the patient's poor baseline condition, as it can lead to rapid onset of hemorrhagic shock or septic shock, posing a significant risk. Therefore, symptomatic treatment is necessary to control bleeding and abdominal infection, and once the patient's condition stabilizes, surgery can be performed to address the residual gastric fistula and intra-abdominal blood vessels. After three surgical procedures to manage abdominal infection, anastomotic fistula, and bleeding, there is a persistent risk of ruptured vessel due to long-term abdominal infection and surgical trauma. Following the management of the primary symptoms, it is crucial to promptly perform digital subtraction angiography to identify the site of vessel rupture or weakness and provide timely intervention, which can yield beneficial treatment outcomes. However, embolization for hemostasis has limitations and is generally more effective for arterial bleeding, while its efficacy for venous bleeding is suboptimal [11]. In this case, the patient did not undergo digital subtraction angiography in a timely manner after the stabilization of their condition but rather underwent the examination 20 days after the onset of symptoms. Although the intervention was performed promptly, avoiding further deterioration, it also increased the patient's risks. This experience provides valuable lessons for the management of similar diseases in the future, in order to prevent the recurrence of the same mistakes.

In this case, both surgery and arterial embolization are indispensable. The simultaneous implementation of these two treatment modalities enhances the success rate in treating delayed massive bleeding following gastric cancer surgery, reduces patient suffering, and prolongs survival. The patient’s ability to be cured and discharged in this case can be attributed to a multidisciplinary approach, with tumor surgery as the main driver and multiple consultations with departments such as vascular surgery, gastroenterology, anesthesia, and radiology. Ultimately, a relatively comprehensive treatment plan was developed, and with the assistance of a multidisciplinary team, the patient's condition was stabilized and they were able to be discharged smoothly.

## Conclusion

In cases of unexplained delayed bleeding following gastric cancer surgery, prompt identification and determination of the underlying causes are crucial. A multidisciplinary approach should be adopted to develop a systematic treatment plan at an early stage. When the patient's condition permits, digital subtraction angiography should be performed promptly to precisely locate the source of bleeding and initiate appropriate interventions.

## Data Availability

All data generated or analysed during this study are included in this published article.

## Abbreviations

CT: Computed tomography
EGD: Esophagogastroduodenoscopy
